# Cognitive Outcomes After Cochlear Implantation in Older Adults: A Narrative Review of Current Evidence, Mechanisms, and Long-Term Perspectives

**DOI:** 10.3390/audiolres16030088

**Published:** 2026-06-06

**Authors:** Luigi Falchetta, Alfonso Scarpa, Mario Carucci, Matteo Calvanese, Luisa Savignano, Antonella Bisogno, Carla De Santis, Arianna Montanino, Caterina Criscuoli, Francesco Antonio Salzano

**Affiliations:** 1 Department of Medicine and Surgery, University of Salerno, 84081 Baronissi, Italy; 2 A.O.U. San Giovanni di Dio e Ruggi d’Aragona, 84131 Salerno, Italy

**Keywords:** cochlear implant, cognitive decline, hearing loss, dementia, older adults, auditory rehabilitation, executive function, cognitive reserve

## Abstract

**Background:** Hearing loss is highly prevalent among older adults and represents a growing public health burden. Increasing attention has been given to the associations between hearing loss, cognitive decline, and dementia risk. Cochlear implantation is an established intervention for adults with severe-to-profound sensorineural hearing loss who obtain limited benefit from conventional amplification, but its potential cognitive effects remain debated. **Objective:** This narrative review summarizes current evidence on cognitive outcomes after cochlear implantation in adults and older adults, with particular attention to cognitive domains, long-term trajectories, methodological limitations, and clinical implications. **Main findings:** Available evidence suggests that cochlear implantation may be associated with improvement or stabilization of selected cognitive domains, particularly attention, executive function, working memory, and memory. However, findings are heterogeneous across studies, cognitive tools, and follow-up durations. Prospective longitudinal studies using hearing-adapted or visually presented cognitive batteries support the possibility of postoperative cognitive benefit, but the magnitude and durability of this effect vary between individuals and domains. Long-term studies suggest that cognitive improvement may be strongest during the first postoperative years and may later stabilize or attenuate, whereas auditory and quality-of-life benefits appear more consistently sustained. **Conclusions:** Cochlear implantation should be regarded as an established hearing rehabilitation strategy with robust benefits for auditory performance and quality of life, and as a potentially cognition-supportive intervention in selected older adults with severe-to-profound hearing loss. Current evidence does not yet prove that cochlear implantation prevents dementia. Future studies should use standardized hearing-adapted cognitive protocols, longer follow-up, adequate comparison groups, and clinically meaningful cognitive endpoints.

## 1. Introduction

Hearing loss is one of the most common sensory impairments worldwide and represents an increasing public health burden in ageing populations [1]. The Global Burden of Disease 2019 study estimated that approximately 1.57 billion people were living with hearing loss in 2019, and the number of affected individuals is projected to increase substantially by 2050 [1]. This burden is particularly relevant in older adults, in whom age-related auditory decline may affect communication, social participation, emotional well-being, functional independence, and quality of life [1,2,3].

Hearing loss has also been increasingly studied as a factor associated with cognitive decline and dementia risk [4,5,6,7]. Several mechanisms have been proposed to explain this association, including increased listening-related cognitive load, auditory deprivation, reduced social engagement, depression, brain structural and functional changes, and shared age-related vascular or neurodegenerative pathways [5,6,7,8]. These mechanisms are not mutually exclusive, and the relationship between hearing loss and cognition is likely multifactorial rather than explained by a single causal pathway [5,6,7,8].

Cochlear implantation is an established treatment for adults with severe-to-profound sensorineural hearing loss who derive insufficient benefit from conventional acoustic amplification [3,8,9,10]. In older adults, cochlear implantation has been associated with substantial improvements in speech perception, communication ability, and disease-specific quality of life [3,9,10,11,12]. Because cochlear implant recipients often have advanced auditory deprivation, cochlear implantation represents a clinically relevant model for investigating whether restoration of auditory input may influence cognitive trajectories [13,14,15,16,17].

Current evidence suggests that cognitive outcomes after cochlear implantation are heterogeneous and domain-specific [13,14,15,16,17]. Systematic reviews and meta-analyses have reported postoperative improvements in global cognitive screening measures and selected neuropsychological domains, but they also highlight variability in cognitive tests, follow-up duration, study design, and control of practice effects [13,14,15,16,17]. Prospective studies suggest that attention, executive function, working memory, immediate memory, and delayed memory may improve after implantation, although not all domains improve consistently and not all improvements persist over long-term follow-up [9,10,11,12,18,19,20,21,22,23].

The aim of this review is to summarize current evidence on cognitive outcomes after cochlear implantation in adults and older adults, focusing on mechanistic rationale, domain-specific cognitive effects, long-term trajectories, cognition as a predictor of cochlear implant outcomes, methodological limitations, and clinical implications.

## 2. Materials and Methods

This is a narrative review informed by a structured literature search, not a systematic review or meta-analysis.

A structured search of PubMed was performed to identify relevant studies on cochlear implantation, hearing loss, and cognitive outcomes. The search strategy combined terms related to hearing loss, cognitive decline, dementia, and auditory rehabilitation. The potential exclusion of studies indexed only in other databases represents a limitation of this review.

The literature search was conducted in April 2026. Studies published within the preceding 10 years (from 2016 to 2026) were considered eligible.

Initial filters were applied to identify recent reviews, systematic reviews, and meta-analyses. Additional targeted searches were then performed to identify relevant prospective longitudinal, controlled, and long-term follow-up studies specifically addressing cognitive outcomes after cochlear implantation.

Pediatric studies were not included in the core synthesis because developmental neurocognitive outcomes after early auditory rehabilitation are biologically and clinically distinct from cognitive trajectories in postlingually deafened adults and older adults. Conference abstracts, editorials, and articles without full-text availability were also excluded.

Only studies published in English were considered eligible.

Studies on hearing aids and broader hearing rehabilitation were considered when relevant for contextualizing auditory rehabilitation and cognition or for comparison with cochlear implant evidence.

**Figure 1:**
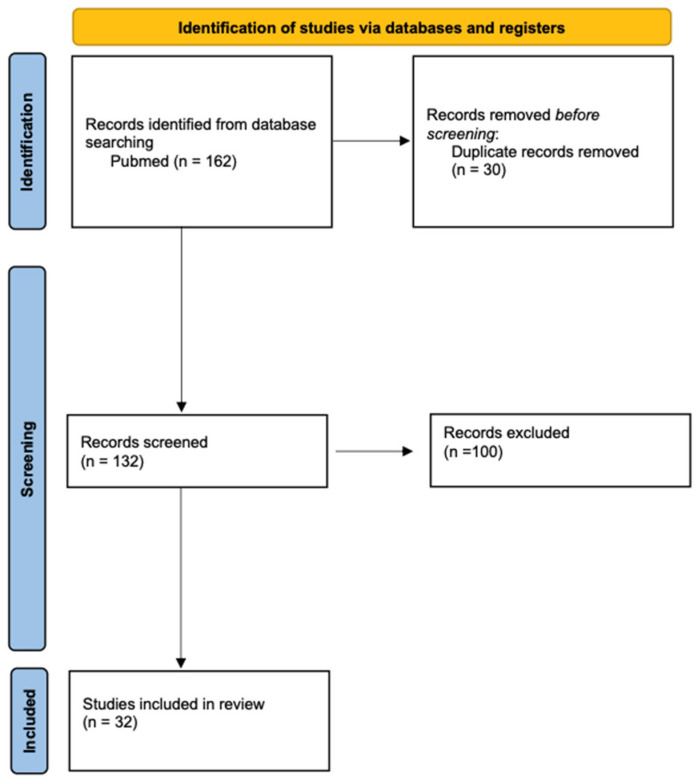
PRISMA-style flow diagram of study selection.

Titles and abstracts were screened for relevance, and full-text articles were assessed when appropriate. A PRISMA-style flow diagram was used only to improve transparency of study identification and selection, without implying that this narrative review followed the full methodological requirements of a systematic review. The study selection process is summarized in Figure 1. No formal risk-of-bias or methodological quality assessment was performed because the aim of this article was to provide a narrative synthesis of current evidence rather than a systematic review or meta-analysis. Nevertheless, major methodological limitations of the included literature, including study design, sample size, follow-up duration, cognitive assessment tools, auditory bias, practice effects, attrition, and control-group availability, were considered narratively when interpreting the findings.

The key studies and reviews addressing cognitive outcomes, cognitive predictors, patient characteristics, cochlear implantation-related details when available, and methodological issues in adult cochlear implant recipients are summarized in Table 1.

CI-related details are reported only when available from the cited studies or clearly extractable from the present narrative synthesis. When specific information on device type, unilateral or bilateral implantation, bimodal stimulation, duration of deafness, rehabilitation intensity, or surgical details was not available, this is indicated as not reported.

## 3. Results

### 3.1. Mechanistic Rationale: Why Cochlear Implantation May Affect Cognition

The cognitive load hypothesis suggests that degraded auditory input forces individuals with hearing loss to allocate greater cognitive resources to speech decoding, leaving fewer resources available for memory, attention, and executive processing [5,6,8]. This mechanism is clinically relevant because speech understanding is not purely auditory but also depends on top-down cognitive processes, including attention, inhibition, working memory, and language processing [8,16].

The sensory deprivation hypothesis proposes that prolonged reduction in auditory input may contribute to cortical reorganization, reduced environmental stimulation, and downstream cognitive vulnerability [5,6,7,8]. Neuroimaging and mechanistic reviews support the possibility that hearing loss may be accompanied by altered auditory cortical processing and compensatory recruitment of higher-order cortical networks [6,7,8]. In this model, restoring auditory input through cochlear implantation may reduce listening effort and increase access to cognitive and social stimulation [5,6,7,8].

Psychosocial mechanisms may further mediate the association between hearing loss and cognition [3,5,6,7]. Hearing loss can reduce communication ability, increase listening fatigue, and promote social withdrawal, loneliness, depression, and reduced cognitive stimulation [3,6,7]. By improving auditory access and communication, cochlear implantation may indirectly support cognition through renewed social participation and better engagement in daily activities [3,8,11,12].

Postoperative rehabilitation may also contribute to cognitive stimulation [9,11,12,21]. Cochlear implant users undergo repeated fitting sessions, auditory training, adaptation to a new auditory signal, and progressive reintegration of auditory input into everyday communication [9,11,12,21]. This rehabilitation pathway may create an enriched sensory and cognitive environment, particularly during the first postoperative months or years [9,11,21,22,23].

The common cause hypothesis remains an important alternative explanation [5,6,7,8]. Hearing loss and cognitive decline may both reflect shared age-related mechanisms, including vascular disease, neurodegeneration, metabolic dysfunction, oxidative stress, frailty, or broader biological ageing [5,6,7,8]. This hypothesis cautions against assuming that cochlear implantation alone can prevent cognitive decline or dementia [8,9,10,11,12,13,14,15,16,17,18,19,20,21,22,23,24,25,26,27,28].

A further issue is that hearing loss can interfere with cognitive testing itself [5,8,13,17]. Cognitive tests that rely on spoken instructions or auditory-verbal stimuli may underestimate cognitive ability before implantation and overestimate cognitive improvement after implantation when audibility improves [5,8,13,17]. For this reason, studies in cochlear implant candidates should use hearing-adapted, visually presented, or non-auditory cognitive instruments whenever possible [9,11,12,17,18,19].

### 3.2. Cognitive Outcomes After Cochlear Implantation

*(i)*  *Cognitive assessment tools and comparability across studies*

Cognitive assessment methods varied substantially across the included literature, limiting direct comparability between studies [11,12,13,14,15]. The reviewed studies used brief global screening tools, hearing-adapted neuropsychological batteries, visually presented computerized tasks, and domain-specific measures of attention, executive function, working memory, verbal fluency, and memory [9,10,11,12,14,15,16,17,18]. Table 2 summarizes the main cognitive instruments reported in the cited studies, their assessed domains, administration modality, suitability for hearing-impaired cochlear implant candidates or users, practical feasibility, and main limitations.

Overall, hearing-adapted or visually presented tools, such as RBANS-H, ALAcog, and Cogstate, are methodologically preferable in cochlear implant candidates because they reduce the risk that poor preoperative audibility is misclassified as cognitive impairment [9,10,11,12,17,18,19]. However, even adapted instruments remain vulnerable to practice effects, differences in follow-up intervals, attrition, and variability in the cognitive domains assessed [10,11,12,13,17,18].

*(ii)*  *Systematic reviews and meta-analyses*

Early systematic evidence suggested that cognitive outcomes after cochlear implantation in older adults were promising but methodologically limited [13]. Claes et al. reported postoperative cognitive improvement in most included longitudinal studies, but the evidence was considered inconclusive because of small samples, lack of control groups, practice effects, and limited use of hearing-adapted cognitive tools [13].

More recent meta-analytic evidence supported postoperative improvement in selected cognitive outcomes, particularly memory/learning, inhibition-concentration, executive function, and verbal memory [14,15]. Pooled analyses reported significant improvements in MMSE, MoCA, and Trail Making Test-B, whereas effects were less consistent across other domains and time points [14,15].

The broader literature on hearing restorative devices also suggests an association between auditory rehabilitation and cognition, but does not prove causality [4,27]. Yeo et al. reported lower long-term cognitive decline and short-term cognitive improvement among users of hearing aids or cochlear implants, although the quality of evidence was low and largely observational [4]. Therefore, hearing interventions should not yet be presented as proven dementia-prevention strategies [27].

*(iii)*  *Prospective studies*

Prospective studies provide more direct evidence on cognitive trajectories after cochlear implantation, although results remain heterogeneous [9,10,11,12,18,19,20,21,22,23]. Hearing-adapted or visually presented batteries have reported improvements mainly in attention, inhibition, working memory, immediate memory, delayed memory, and executive function [9,10,18,19,20]. Among these domains, attention appears relatively robust: in the controlled multicenter study by Mertens et al., cochlear implantation was associated with improvement in RBANS-H total score and particularly in the attention subdomain compared with matched hearing-impaired controls [10].

Other domains show more variable findings. Memory improvements have been reported in several cohorts, but they are less consistently maintained after control-group comparison or long-term follow-up [10,12,18,22,23]. Cognitive reserve and baseline cognitive status may influence postoperative cognitive trajectories, although the COCHLEA study found no evidence that treatment effects varied according to baseline cognitive performance after accounting for regression-to-the-mean effects [11,20,21].

*(iv)*  *Long-term cognitive trajectories*

Long-term evidence remains limited but increasingly informative [11,12,22,23]. Völter et al. reported that improvements in attention, delayed recall, working memory, verbal fluency, and inhibition occurred mainly during the first postoperative year and then remained broadly stable up to approximately 65 months [22]. A subsequent analysis described memory trajectories characterized by early postoperative improvement followed by plateau or decline after approximately two years, supporting a “cognitive booster” model rather than definitive dementia prevention [23].

The COCHLEA study provided prospective data up to 4.5 years and found improvement in executive function and working memory among cochlear implant users, while higher daily device use was associated with earlier and greater cognitive improvement [11]. Conversely, Vandenbroeke et al. reported that global RBANS-H improvement was no longer significant at four years, whereas auditory and disease-specific quality-of-life benefits remained more durable [12]. Taken together, these studies suggest that cognitive benefits may be strongest in the first postoperative years and may later stabilize or attenuate, while auditory and quality-of-life outcomes are more consistently sustained [11,12,22,23]. This pattern supports a cognitive resilience model rather than a generalized cognitive-enhancement model [11,12,14,15,16,17,22,23].

### 3.3. Cognition, Cochlear Implant Performance, and Pre-Existing Cognitive Impairment

Cognition may influence not only postoperative cognitive trajectories but also auditory outcomes after cochlear implantation [14,16]. Speech recognition with a cochlear implant depends on both bottom-up auditory input and top-down cognitive processes, including attention, inhibition, working memory, verbal fluency, and processing speed [14,16].

Amini et al. examined the relationship between cognition and speech recognition outcomes in adult cochlear implant users [16]. Global cognition and inhibition-concentration were the cognitive domains most consistently associated with postoperative speech recognition [16]. Pooled analyses showed a moderate positive association between global cognition and speech recognition, and verbal fluency was also significantly associated with speech outcomes [16]. In contrast, memory and learning measures did not show significant pooled associations with speech recognition in quiet or in noise [16].

Testing conditions may influence the relationship between cognition and cochlear implant outcomes [16]. Speech recognition in quiet is more frequently studied than speech recognition in noise, although speech-in-noise performance is more representative of real-world listening and may place greater demands on attention, inhibition, and working memory [16]. Heterogeneity in speech materials and background noise types limits comparisons across studies [16].

Patients with pre-existing cognitive impairment represent a clinically important subgroup of cochlear implant candidates [24,25]. Dawes et al. reviewed 13 studies including 222 cochlear implant recipients with cognitive impairment, mild cognitive impairment, or dementia [24]. Available evidence suggested that speech recognition generally improved after implantation in cognitively impaired recipients, although benefits may be smaller than in cognitively healthy users and may depend on severity and progression of cognitive impairment [24].

Available data do not clearly show higher rates of adverse events or cochlear implant non-use among recipients with cognitive impairment, but long-term device use remains insufficiently studied [24]. Evidence for direct cognitive improvement in patients with established cognitive impairment is weaker than evidence for auditory benefit [24,25]. Mamo et al. found that hearing loss treatment in older adults with cognitive impairment may improve communication, quality of life, and dementia-related behavioural symptoms, but robust evidence for direct cognitive improvement was insufficient [25].

Cognitive impairment should not be used as an automatic exclusion criterion for cochlear implantation [20,24]. Instead, cognitive status should guide counselling, caregiver involvement, rehabilitation planning, device-management support, and follow-up intensity [20,24,25]. In cognitively impaired patients, realistic goals may include improved speech recognition, communication with caregivers, participation in daily activities, and quality of life, rather than guaranteed cognitive recovery [24,25].

## 4. Discussion

The present review highlights that cochlear implantation is associated with domain-specific cognitive effects rather than global cognitive enhancement. The heterogeneity of findings across studies reflects both the biological complexity of the hearing-cognition relationship and the substantial methodological variability in the field. The following section addresses the main limitations of available evidence and outlines priorities for future research.

This cautious interpretation is further supported by evidence from the hearing aid literature. In a systematic review including 17 longitudinal studies and 3526 participants, Sanders et al. found substantial heterogeneity in study design, follow-up duration, and cognitive outcome measures; the included studies used 50 different cognitive tests, preventing meta-analysis and limiting direct comparability across studies [29]. Their results suggested that, if a cognitive benefit of hearing aids exists, it may be most evident in executive function, whereas effects on complex attention, language, learning, and memory were less consistent or largely absent [29]. Importantly, most included studies had relevant methodological limitations, including short follow-up, high risk of bias, limited exposure assessment, and cognitive tests not always designed for hearing-impaired populations [29]. These findings are relevant to cochlear implant research because they reinforce the need to interpret postoperative cognitive changes as domain-specific, potentially test-dependent, and vulnerable to methodological bias rather than as evidence of generalized cognitive restoration.

Similar conclusions were reached by Yang et al., who systematically reviewed 15 studies evaluating the effects of hearing aids on cognitive function in middle-aged and older adults with hearing loss, including five randomized controlled trials and ten non-randomized intervention studies [30].

These observations are consistent with the broader hearing rehabilitation literature, which supports early identification and timely management of hearing loss as part of an integrated strategy addressing auditory function, cognitive health, mood, social participation, and quality of life [31].

### 4.1. Rehabilitation Considerations in Older Cochlear Implant Recipients

Postoperative rehabilitation and follow-up are important components of cochlear implant care in older adults and may influence auditory outcomes, daily device use, communication, and broader functional benefits [3,11,12,24,25]. In the COCHLEA study, higher daily cochlear implant use was associated with earlier and greater cognitive improvement, supporting the importance of sustained device use during follow-up [11]. In older adults, especially those with cognitive impairment, rehabilitation planning may need to incorporate realistic counselling, caregiver involvement when appropriate, support for device management, and close follow-up, although evidence on specific support needs and caregiver-related outcomes remains limited [24,25,27]. Expected benefits should therefore be framed primarily in terms of speech recognition and communication, while broader outcomes such as quality of life, daily functioning, social participation, behavioural symptoms, and caregiver burden require further study [24,25]. Cognitive impairment should not be considered an automatic contraindication to cochlear implantation, but it should guide counselling, rehabilitation planning, and follow-up organization [20,24].

### 4.2. Methodological Limitations and Future Directions

The interpretation of cognitive outcomes after cochlear implantation is limited by recurrent methodological issues [13,14,15,16,17]. These include auditory bias in cognitive testing, practice effects, heterogeneous cognitive batteries, small samples, short follow-up, attrition, lack of appropriate control groups, and incomplete adjustment for confounders [11,12,13,14,15,16,17]. These limitations may either overestimate or underestimate the true cognitive effect of cochlear implantation [13,14,15,16,17].

Auditory bias is a central concern because many cognitive tools rely on spoken instructions or verbally presented stimulations [13,14,17]. In patients with severe-to-profound hearing loss, poor audibility may reduce baseline test performance independently of true cognitive ability [13,14,17]. After implantation, improved access to spoken instructions or test material may improve scores even if underlying cognition has not changed [13,14,17].

Hearing-adapted and visually presented tools reduce this problem but do not eliminate all bias [9,10,11,12,17,18,19]. The RBANS-H provides written instructions and audiovisual presentation for selected subtests and has been used in several cochlear implant studies [10,12,18]. The ALAcog battery and Cogstate battery are visually presented and reduce dependence on auditory instruction [9,10,19,21,23]. However, repeated testing can still produce practice effects even with adapted instruments or alternate forms [10,12,13,17,18].

Appropriate comparison groups are needed but difficult to establish [10,12,17]. Matched patients with severe-to-profound hearing loss who do not receive cochlear implants would provide the most relevant comparison, but withholding treatment for long periods may be ethically and practically problematic [10,12,17]. External comparison cohorts, such as SHARE, HRS, ELSA, and AIBL, provide useful context, but they differ from cochlear implant cohorts in hearing status, cognitive assessment methods, baseline risk factors, education, health status, and motivation [11,22,23].

Sample size, attrition, and follow-up duration remain major limitations [9,11,12,13,15,18,19]. Several studies have modest baseline samples and substantially smaller long-term follow-up samples [9,11,12,18,19]. Since cognitive decline and dementia develop over years, short-term improvements after implantation cannot establish dementia prevention [11,12,22,23,27]. Long-term studies suggest that some cognitive benefits may stabilize or attenuate after the first postoperative years, while auditory and quality-of-life benefits are more consistently sustained [11,12,22,23].

Future studies should use standardized, hearing-adapted, domain-specific cognitive protocols [10,12,17,18]. Instruments such as RBANS-H, visually presented Cogstate tasks, ALAcog, MoCA-HI, HI-ACE-III, and other non-auditory or hearing-adapted assessments may reduce auditory bias and improve comparability [9,10,17,18,19]. Global cognitive screening tools alone are insufficient because they may miss domain-specific changes and may include auditory-verbal components [14,17].

Future research should also include objective device-use measures, rehabilitation variables, and broader confounder adjustment [11,12,17,21]. Relevant variables include age, education, baseline cognitive reserve, duration of deafness, residual hearing, bimodal hearing status, device use, rehabilitation intensity, depression, social isolation, cardiovascular risk factors, diabetes, smoking, alcohol use, frailty, and genetic risk [11,12,17,21]. Data logging may help clarify whether sustained auditory stimulation influences cognitive trajectories [11].

Multimodal approaches may help distinguish true neurocognitive change from improved audibility or practice effects [8,17]. Future studies could combine neuropsychological testing with electrophysiology, neuroimaging, biomarkers, ecological measures of communication, patient-reported outcomes, and computational approaches [8,11,12,17]. Event-related potentials, including visual and auditory paradigms, may be useful, although auditory evoked responses may be affected by implant-related artifacts [8,17].

Future work should avoid overreliance on dementia prevention unless studies are specifically designed to evaluate that endpoint [11,12,27]. The more appropriate near-term goal is to determine whether cochlear implantation supports cognitive resilience, slows decline in selected domains, or improves performance through reduced listening effort and increased engagement [11,12,22,23,27]. Establishing dementia-risk reduction would require larger samples, longer follow-up, rigorous cognitive endpoints, and careful control of confounding [11,12,27].

## 5. Conclusions

Cochlear implantation is an established and effective intervention for adults with severe-to-profound sensorineural hearing loss, with robust benefits for speech perception, communication, and disease-specific quality of life [3,9,10,11,12]. Current evidence also suggests that cochlear implantation may improve or stabilize selected cognitive domains in older adults, particularly attention, executive function, working memory, inhibition, and memory-related functions [9,10,11,12,14,15,16,17,18,19,20,21,22,23].

The cognitive effects of cochlear implantation are not uniform across studies, patients, or domains [9,10,11,12,14,15,16,17,19,20,21,22,23]. Short- and medium-term studies often report postoperative cognitive improvement, while long-term studies suggest that cognitive benefits may stabilize or attenuate over time [11,12,22,23]. Auditory and quality-of-life benefits appear more consistently sustained than global cognitive improvements [11,12].

The evidence does not yet prove that cochlear implantation prevents dementia [9,24,25]. Observational designs, small long-term samples, heterogeneous cognitive tools, incomplete control of confounders, practice effects, and limited clinically defined dementia endpoints prevent firm causal conclusions [11,12,13,14,15,16,17,27]. Therefore, cochlear implantation should be described as a potentially cognition-supportive hearing intervention rather than an established anti-dementia treatment [11,12,27].

From a clinical perspective, cognitive impairment should not automatically exclude older adults from cochlear implantation [20,24]. Instead, cognitive status should guide counselling, caregiver involvement, rehabilitation planning, and follow-up intensity [20,24,25]. The most balanced interpretation is that cochlear implantation may contribute to cognitive resilience in selected older adults with severe-to-profound hearing loss while providing clear and durable benefits for auditory function and quality of life [10,11,12,22,23].

## Figures and Tables

**Table 1:** Key studies and reviews evaluating cognitive outcomes, cognitive predictors, patient characteristics, cochlear implantation-related details, and methodological issues in adult cochlear implant recipients.

Study	Design	Population/Sample	CI-Related Details Reported	Comparator	Follow-Up	Cognitive Assessment/Focus	Main Findings	Key Limitations/Interpretative Issues
Vandenbroeke et al., 2025 [8]	Narrative review	Older adult cochlear implant population	Not applicable; review-level evidence	Not applicable	Not applicable	Current evidence and future perspectives on cognition after cochlear implantation	Summarized evidence on cognition after cochlear implantation in older adults, emphasizing methodological heterogeneity, auditory bias in cognitive testing, and need for standardized hearing-adapted protocols.	Narrative design; no quantitative synthesis; conclusions depend on heterogeneity of included studies.
Claes et al., 2018 [13]	Systematic review	166 older cochlear implant recipients across 6 longitudinal studies	CI details varied across included studies; specific device/surgical characteristics not summarized in the present review	Limited or variable across included studies	Variable	Multiple cognitive tests	Most included studies reported postoperative cognitive improvement, but evidence was considered inconclusive.	Small samples, lack of control groups, practice effects, and limited use of hearing-adapted cognitive tools.
Amini et al., 2023 [14]	Systematic review and meta-analysis	Adult cochlear implant users	CI details varied across included studies; specific implantation characteristics not summarized in the present review	Variable across included studies	Variable	MMSE, MoCA, Trail Making Test, and other cognitive measures	Approximately half of reported cognitive outcomes improved after cochlear implantation; pooled analyses showed significant improvements in MMSE, MoCA, and Trail Making Test-B.	Heterogeneous cognitive tools, follow-up intervals, and outcome definitions; pooled estimates limited by variability of primary studies.
An et al., 2023 [15]	Systematic review and meta-analysis	648 older cochlear implant recipients from 20 longitudinal studies	CI details varied across included studies; specific implantation characteristics not summarized in the present review	Variable across included studies	6–12 months	Global cognition, executive function, verbal and non-verbal memory	Executive function and verbal memory improved within 6 months; global cognition and non-verbal memory showed clearer improvement at 12 months.	Limited by heterogeneity of included studies, short-to-medium follow-up, and variable cognitive outcome measures.
Amini et al., 2024 [16]	Scoping review and meta-analysis	Adult cochlear implant users	CI details varied across included studies; specific implantation characteristics not summarized in the present review	Variable across included studies	Variable	Relationship between cognition and cochlear implant speech outcomes	Global cognition and inhibition-concentration were most consistently associated with postoperative speech recognition; verbal fluency also showed significant association.	Heterogeneity in cognitive measures, speech materials, and quiet versus noise testing conditions.
Villarreal-Garza & Callejón-Leblic, 2025 [17]	Scoping review	Studies evaluating neuropsychological assessment after cochlear implantation	Not applicable; focused on assessment tools rather than implantation variables	Not applicable	Not applicable	Cognitive assessment tools and methodological issues	Highlighted heterogeneity of cognitive instruments and need for hearing-adapted, visually presented, or non-auditory cognitive assessment tools.	Scoping review design; does not provide formal effect estimates; emphasizes methodological mapping rather than outcome synthesis.
Claes et al., 2018 [18]	Prospective longitudinal cohort study	Severely hearing-impaired older adults undergoing cochlear implantation	20 older adults, median age 71.5 years, undergoing unilateral CI; post-activation auditory rehabilitation offered.	No control group reported in the present narrative synthesis	12 months	RBANS-H	Significant improvement in total cognitive score after cochlear implantation, mainly driven by immediate and delayed memory.	Preliminary cohort evidence; absence of control group limits separation of treatment effect from practice effects or natural variability.
Völter et al., 2018 [9]	Prospective cohort study	Aging cochlear implant recipients	60 adults aged 50–84 years with severe-to-profound bilateral hearing impairment undergoing unilateral CI.	No control group reported in the present narrative synthesis	6–12 months	ALAcog visually presented cognitive battery	Improvements in attention, inhibition, working memory, and delayed recall at 6 months; verbal fluency improved later at 12 months.	Cohort design; limited control for practice effects; comparability restricted by use of a specific cognitive battery.
Sarant et al., 2019 [19]	Prospective international longitudinal study	Older adults with severe-to-profound hearing loss undergoing cochlear implantation	Older adults undergoing CI; detailed device/surgical characteristics not reported in the present summary	No control group reported in the present narrative synthesis	18 months	Visually presented Cogstate battery	Speech perception, communication ability, and quality of life improved; cognition remained stable in most participants, with executive function improving in a subgroup.	Subgroup findings require caution; absence of a matched untreated control group limits causal interpretation.
Mertens et al., 2021 [10]	Prospective longitudinal controlled multicenter study	Older cochlear implant users compared with matched hearing-impaired controls	Older CI users; detailed device/surgical characteristics not reported in the present summary	Matched hearing-impaired controls without cochlear implantation	14 months	RBANS-H	CI was associated with improvement in RBANS-H total score and particularly in attention compared with controls; memory improvements were less robust after control comparison.	Stronger design due to control group, but still limited by follow-up duration and potential residual confounding.
Gurgel et al., 2022 [20]	Prospective cohort study	Adults aged ≥65 years undergoing cochlear implantation	Adults ≥65 undergoing CI; detailed device/surgical characteristics not reported in the present summary	No control group reported in the present narrative synthesis	12 months	Verbal and visually based cognitive tasks	Improvements in attention, executive function, verbal learning and memory, and working memory; benefits appeared marked among participants with preoperative cognitive impairment.	Cohort design; findings in cognitively impaired participants require cautious interpretation because regression to the mean and practice effects may contribute.
Völter et al., 2022 [21]	Prospective cohort/cognitive reserve study	Adult cochlear implant recipients	Adult CI recipients; detailed device/surgical characteristics not reported in the present summary	Not reported in the present narrative synthesis	Postoperative follow-up	Cognitive reserve and cognitive performance measures	Cognitive reserve was associated with cognitive performance before and after implantation; greater cognitive improvement was observed in recipients with lower cognitive reserve and poorer baseline cognition.	Cognitive reserve effects may be influenced by baseline differences, regression to the mean, and heterogeneity in patient characteristics.
Völter et al., 2022 [22]	Longitudinal follow-up study	Adult cochlear implant recipients	Adult CI recipients; detailed device/surgical characteristics not reported in the present summary	Not reported in the present narrative synthesis	Up to approximately 65 months	Multiple cognitive domains	Improvements in attention, delayed recall, working memory, verbal fluency, and inhibition, with most gains occurring during the first postoperative year and then remaining broadly stable.	Long-term follow-up is informative but may be affected by attrition, practice effects, and lack of robust external control.
Völter et al., 2023 [23]	Longitudinal trajectory study	Middle-aged and older people with hearing loss using cochlear implants	CI users; detailed device/surgical characteristics not reported in the present summary	Not reported in the present narrative synthesis	Longitudinal follow-up	Memory trajectories	Memory trajectories suggested early postoperative improvement followed by plateau or decline after approximately two years, supporting a “cognitive booster” rather than dementia-prevention interpretation.	Memory-specific trajectories may not generalize to all cognitive domains; long-term interpretation limited by cohort design and attrition.
Sarant et al., 2024 [11]	Prospective longitudinal cohort study/COCHLEA study	101 CI participants, older adults, with hearing loss and cochlear implants compared with AIBL participants	CI use and daily device-use analysis reported; higher daily use, at least 14 h/day, associated with earlier and greater cognitive improvement	AIBL comparison cohort	4.5 years	Multiple cognitive domains; device-use analysis	CI users showed improvement in executive function and working memory and stability in other domains; AIBL participants worsened in attention and psychomotor function; higher device use associated with earlier and greater cognitive improvement.	External comparison cohort may differ from CI cohort in baseline characteristics, hearing status, motivation, and assessment context; device-use association does not prove causality.
Vandenbroeke et al., 2025 [12]	Prospective longitudinal study	Older adults undergoing cochlear implantation	Older adults undergoing CI; detailed device/surgical characteristics not reported in the present summary	No control group reported in the present narrative synthesis	Up to 4 years	RBANS-H	RBANS-H total score, immediate memory, attention, and delayed memory improved at 1 year; global cognitive improvement was no longer significant at 4 years, whereas auditory and quality-of-life benefits remained more durable.	Long-term findings limited by attrition and absence of a control group; global cognitive benefit may attenuate over time.
Dawes et al., 2024 [24]	Scoping review	Cochlear implant recipients with cognitive impairment, mild cognitive impairment, or dementia	CI recipients with cognitive impairment; specific implantation details varied and are not summarized in the present review	Variable across included studies	Variable	Auditory and cognitive outcomes in cognitively impaired recipients	Speech recognition generally improved after cochlear implantation in cognitively impaired recipients, although benefits may be smaller than in cognitively healthy users; cognitive impairment should not be considered an automatic exclusion criterion.	Evidence base is limited; data on long-term device use, non-use, adverse events, caregiver outcomes, and cognitive outcomes remain insufficient.
Mamo et al., 2018 [25]	Systematic review	Older adults with cognitive impairment receiving hearing loss treatment	Broader hearing loss treatment; not limited to cochlear implantation	Variable across included studies	Variable	Hearing loss treatment, cognition, communication, quality of life, behavioral symptoms	Hearing loss treatment may improve communication, quality of life, and dementia-related behavioral symptoms, but evidence for direct cognitive improvement remained insufficient.	Broader hearing rehabilitation review; applicability to CI-specific outcomes is indirect.
Carasek et al., 2022 [26]	Systematic review	Older adults using cochlear implants and/or hearing aids	Includes CI and/or hearing aid users; specific CI details not summarized in the present review	Variable across included studies	Variable	Cognition after auditory rehabilitation	Suggested possible cognitive benefit from hearing rehabilitation, including cochlear implants and hearing aids, but emphasized heterogeneity and need for stronger prospective evidence.	Mixed-device evidence; heterogeneity limits CI-specific conclusions.

**Table 2:** Cognitive assessment tools used in studies evaluating cognitive outcomes after cochlear implantation in adults and older adults.

Cognitive Tool/Battery	Main Cognitive Domains Assessed	Administration Modality	Hearing-Adapted or Visually Presented?	Practical Feasibility	Main Limitations in Older CI Candidates/Users	Use in the Cited Literature *
MMSE	Global cognition; orientation; memory; attention; language	Mostly oral, with some written/visuospatial components	No	Very easy to administer; widely available; familiar to clinicians	Limited sensitivity to mild cognitive impairment; ceiling effects; potential auditory and language bias in severely hearing-impaired patients	Reported in meta-analytic evidence [14]
MoCA	Global cognition; executive function; attention; memory; language; visuospatial function	Oral and paper-based tasks	No, unless adapted	Easy to administer; more sensitive than MMSE for mild cognitive impairment	Auditory-verbal components may underestimate cognition before implantation; language and education effects; not CI-specific	Reported in meta-analytic evidence [14]
MoCA-HI	Global cognition adapted for hearing-impaired individuals	Written/visual adaptation of MoCA	Yes	Clinically feasible; useful screening option for hearing-impaired adults	Less frequently used in CI outcome studies; mainly a screening tool rather than a full neuropsychological battery	Discussed as a future methodological option [17]
RBANS-H	Immediate memory; visuospatial/constructional function; language; attention; delayed memory	Hearing-adapted neuropsychological battery	Yes	More comprehensive than MMSE/MoCA; suitable for hearing-impaired adults; used in longitudinal CI studies	Longer than screening tools; practice effects remain possible; requires trained administration and interpretation	At least 3 primary studies [10,12,18]
ALAcog battery	Attention; inhibition; working memory; delayed recall; verbal fluency	Visually presented cognitive battery	Yes	Reduces auditory bias; allows domain-specific assessment	Less widely available than standard screening tools; comparability with other studies may be limited	Reported in Völter et al. and related longitudinal studies [9,21,22,23]
Cogstate battery	Executive function; attention; psychomotor speed; visual learning/memory; working memory	Computerized, visually presented tasks	Yes	Standardized and repeatable; suitable for longitudinal studies; reduces auditory dependence	Requires familiarity with computerized testing; visual/motor speed may influence performance in older adults	At least 2 studies from the Sarant/COCHLEA cohort [11,19]
Trail Making Test A	Processing speed; visual scanning; attention	Paper-based visual task	Yes, largely non-auditory after instructions	Quick and easy; useful for attention/processing speed	Influenced by visual acuity, motor speed, education, and familiarity with paper-pencil tasks	Reported in meta-analytic evidence [14]
Trail Making Test B	Executive function; cognitive flexibility; set-shifting; attention	Paper-based visual task	Yes, largely non-auditory after instructions	Quick and clinically familiar; useful executive measure	Influenced by processing speed, visual acuity, motor function, education; practice effects possible	Reported in meta-analytic evidence [14]
Verbal fluency tests	Lexical retrieval; semantic memory; executive control; language	Oral verbal production task	Partially; task output is oral but not dependent on hearing stimuli after instructions	Simple, fast, and widely used	Language, education, and speech production effects; limited comparability across languages; not purely non-auditory	Reported in studies/reviews evaluating CI outcomes [9,14,16,22]
Inhibition/concentration tasks	Inhibitory control; selective attention; executive function	Variable; paper-based or computerized depending on study	Variable	Useful for executive-domain assessment; relevant to speech perception outcomes	Heterogeneous tasks across studies; limited standardization; may reduce comparability	Reported in systematic/meta-analytic evidence [14,16]
Working memory tasks	Short-term memory; manipulation of information; attentional control	Variable; visual, oral, or computerized depending on study	Variable	Relevant to speech understanding and CI outcomes	Test modality varies substantially; auditory-verbal versions may be biased in hearing-impaired patients	Reported across longitudinal studies and reviews [9,11,15,17]
Memory-specific tests/subtests	Immediate memory; delayed memory; verbal and/or visual learning	Variable depending on battery	Variable; better when visual/hearing-adapted	Important because memory is one of the most frequently investigated domains	Verbal memory tests may be confounded by audibility; visual memory tests may not be directly comparable with auditory-verbal memory measures	Reported across several longitudinal studies and reviews [11,12,15,18,20,23]

* Counts should be interpreted cautiously because some cited systematic reviews and meta-analyses report cognitive tools across primary studies that are not all individually discussed in the present narrative review.

## Data Availability

No new data were created or analyzed in this study.

## Abbreviations

The following abbreviations are used in this manuscript:

CI	Cochlear Implant
HL	Hearing Loss
SNHL	Sensorineural Hearing Loss
NCIQ	Nijmegen Cochlear Implant Questionnaire
